# In Vitro Gastrointestinal Digestion and Colonic Fermentation of High Dietary Fiber and Antioxidant-Rich Mango (*Mangifera indica* L.) “Ataulfo”-Based Fruit Bars

**DOI:** 10.3390/nu11071564

**Published:** 2019-07-11

**Authors:** Luz M. Hernández-Maldonado, Francisco J. Blancas-Benítez, Victor M. Zamora-Gasga, Alicia P. Cárdenas-Castro, Juscelino Tovar, Sonia G. Sáyago-Ayerdi

**Affiliations:** 1 Tecnológico Nacional de México/Instituto Tecnológico de Tepic, Av. Tecnológico 2595, Nayarit, CP 63175 Tepic, Mexico; 2 Department of Food Technology, Engineering and Nutrition, Lund University, P.O. Box 124, SE-221 00 Lund, Sweden

**Keywords:** mango, fruit-based bars, in vitro digestion, bioaccessibility, phenolic compounds, antioxidant capacity

## Abstract

Mango (*Mangifera indica* L.) is a tropical fruit which is considered to be a source of dietary fiber (DF) and phenolic compounds (PCs). In this study, high DF mango-based fruit bars were developed from whole mango (peel and pulp). The bars were evaluated for their nutritional composition, the bioaccesibility of PCs during gastrointestinal digestion, and the PCs metabolites profile after in vitro colonic fermentation. The amount of DF in a 30 g portion of mango bars was 9.5 g, i.e., 35% of the recommended daily intake. Phenolic acids such as gallic acid; cinnamic acids, such as ferulic, coumaric, and caffeic acids; flavonoids such as quercertin; and xanthones such as mangiferin and mangiferin gallate, were identified as the main PCs in the bars. The antioxidant capacity associated with the PCs profile, together with the high DF content are indicative of the potential functional features of these natural fruit bars. The bioaccesibility of PCs in the mango bar was 53.78%. During fermentation, the PCs were bioconverted mainly to hydroxyphenolic acids and the main short-chain fatty acid produced was acetic acid. The xanthone norathyriol was identified after 12 h of fermentation. This study on the digestion and colonic fermentation of mango-based bars using in vitro models provides hints of the potential physiological behavior of PCs associated with DF, which constitutes relevant information for further development of natural and health-promoting fruit-based bars.

## 1. Introduction

Mango (*Mangifera indica* L) is one of the most consumed tropical fruits worldwide. In Mexico, the *per capita* consumption of the fruit is 12 kg/person/year [1]. “Ataulfo” is a Mexican mango variety in high demand in the international market because of its unique sensory properties which include firm consistency, sweet drupe, low acidity, and intense aroma [2]. Unfortunately, most of the harvested fruit is not exported, particularly due to its amply variable size [1]. As a matter of fact, mango is one of the most wasted fruits in Mexico [3]. There is interest in minimizing mango losses and by incorporating these fruits into other edible products is a suitable option. Snacks are a secondary form of nutrient intake and they can be as important as main meals in nutritional terms. Snacking behaviors/patterns have been related to obesity in children and adolescents [4], as well as in adults [5]. The incidence of obesity is increasing, and it can be promoted or halted via dietary choices. Nevertheless, it should be clearly stated that snacking per se does not directly correlate with obesity, but appropriate choices must be made in order to avoid long-term detrimental health effects. Healthier snacks with no added sugars or preservatives and with natural flavors are gaining in popularity, and fruit-based snacks could be an attractive option [6,7].

The “Ataulfo” variety contains higher levels of phenolic compounds (PCs), specifically phenolic acids and flavonoids with antioxidant capacity (AOX), than other mango varieties [8] and it also contains important amounts of dietary fiber (DF). The DF in Ataulfo mango peel and paste can reach up to 40% and 14%, respectively, with a good soluble dietary fiber (SDF)/total dietary fiber (TDF) ratio [9]. Nowadays, industry and consumers are looking for novel sources of DF, and fruits and vegetables exhibit very good qualities, not only for their non-starch polysaccharide composition but also for the PCs that are linked to the DF. The PCs that are released from the DF (or from the food matrix) in the digestive tract are considered bioaccessible [10]. However, this concept does not define the amount of PCs that is absorbed, and thus reach potential target organs or cells to exert their bioactivity, as reported for the anti-inflammatory effects of digested bioactive-rich foods on cultured cells [11,12]. Hence, there is interest in identifying, in a quantitative manner, the PCs that are bioaccessible in the gastrointestinal tract, and therefore are able to cross the intestinal barrier, as a result of their release from the DF [13]. In addition, the non-digested or non-absorbed PCs that remain bound to the DF reach the colon. The DF and PCs can be available as a substrate to be partially or completely fermented by the gut microbiota. Fermentable DF produces short chain fatty acids (SCFA), which provide positive health effects, such as anti-inflammatory and/or anti-proliferative properties, that could prevent irritable bowel syndrome or colon cancer [14]. The PCs can modulate the composition of gut microbiota and thus increase the release of metabolites that are often more active and better absorbed than native PCs. A recently emerged concept, the “three P for intestinal health”, includes probiotics, prebiotics (DF), and PCs, and promotes PCs at the same biological level as prebiotics [15]. Therefore, the aim of the present study was to prepare bars from a blend of the pulp and peel of Ataulfo mango and to evaluate the bioaccesibility of their PCs during the gastrointestinal digestion. In addition, the PCs metabolites and short-chain fatty acids (SCFA) produced after in vitro colonic fermentation of the non-bioaccessible fraction of the bars were also investigated.

## 2. Materials and Methods

### 2.1. Preparation of Mango-Based Bars

Mangoes (cv. Ataulfo) were purchased from the local market in Tepic, Nayarit State, México. The fruits were washed with tap water, disinfected (Biopur, Mexico) and sliced (peel with pulp) 3 mm thick (Tor-Rey, R-300, México). Fruit slices were dried in an oven with air-forced convection at 70 °C for 22 h; (Scorpion Scientific, A-52055, Mexico). The dried slices were ground in a food processor (NutriBullet, NBR-0804B, Los Angeles, CA, USA). To prepare the mango-based bars, the ground fruit was molded with a binding agent (Nutriose FB, Tecnovam, Mexico), and the bars were dried at 60 °C for 3 h (Scorpion Scientific, A-52055, Mexico). The bars were prepared without any sweetening additive or preservative. The bar samples were freeze-dried (FreeZone 6, Labconco, Kansas City, MO, USA), and subsequently ground (NutriBullet), sieved (0.5 microns), and stored in sealed bags at −20 °C until analysis.

### 2.2. Nutritional Composition of Mango-Based Bars

Moisture (925.10 method), ash (method 923.03), fat (920.39 method), and protein (920.87 method, protein conversion factor N × 6.25) were analyzed according to AOAC (1990) methods. Total soluble carbohydrates were measured using the phenol sulfuric method [16]. Soluble dietary fiber (SDF), insoluble dietary fiber (IDF), and total dietary fiber (TDF) were analyzed by the AOAC enzymatic-gravimetric method (method 991.42) modified [17] and the results were expressed as g/100 g dry weight (DW). 

### 2.3. Total Soluble Polyphenols (TSP), Hydrolyzable Polyphenols (HP) Content, and Antioxidant Capacity (AOX) in Mango-Based Bars

The samples were submitted to a double aqueous-organic extraction with an acidic methanol solution (50:50 *v*/*v*) and acetone-water solution (70:30 *v*/*v*) [18]. Afterwards, the samples were centrifuged to recover the supernatant, which was analyzed for TSP content using the Folin–Ciocalteu reagent (F9252, Sigma-Aldrich, St. Louis, MO, USA) as described by Montreau [19] with modifications. The absorbance was measured at 750 nm using a 96 well microplate reader (Bio-Tek^®^, Synergy, HT, USA) and Gen5 software. Gallic acid was used as a standard (0.0125–0.2 mg/mL, R^2^ ≥ 0.9997), and the results were expressed as mg of gallic acid equivalents, i.e., g GAE/100 g dry weight (DW). The residue obtained from the aqueous organic extraction was used to evaluate the HP content following the methodology proposed by Hartzfeld et al. [20]. A standard curve was prepared with gallic acid (0.0–0.2 mg gallic acid/mL) and the results were expressed as gallic acid equivalents (g GAE/100 g DW). The AOX was analyzed, in the supernatants of the aqueous-organic extracts, using two common colorimetric methods: the 2,2′-azino-bis (3-ethylbenzthiazoline-6-sulfonic acid) (ABTS) assay and the ferric reducing antioxidant power (FRAP) assay. The ABTS assay was performed as described by Re et al. [21], and the FRAP assay was performed as described by Benzie and Strain [22]. Trolox (6-hydroxy-2,5,7,8-tetramethylchroman-2-carboxylic acid) was used as a standard, and AOX was expressed as μmol of Trolox equivalents (TE)/100 g DW of sample.

### 2.4. Identification of PCs by HPLC-DAD-MS Analysis of the Mango-Based Bars

In addition, in order to quantify the TSP data, the PCs were chromatographically identified by HPLC using an Agilent 1260 Series Instrument (Agilent Technologies, Santa Clara, CA, USA) equipped with a UV–Vis diode array detector DAD (Agilent Technologies G4212-60008, Santa Clara, CA, USA). The samples were injected (10 μL, flow rate of 0.4 mL/min) into a Poroshell 120 EC-C18 column (4.6 mm × 150 mm, particle size of 2.7 μm) (Agilent Technologies). The gradient elution was carried out using water containing 0.1% trifluoracetic acid (302031, Sigma-Aldrich, St. Louis, MO, USA) as solvent A and acetonitrile (Sigma Aldrich, St. Louis, MO, USA) as solvent B as follows: 0 min, 5% B; 10 min, 23% B; 15 min, 50% B; 20 min, 50% B; 23 min, 100% B; 25 min, 100% B; 27 min, 5% B; and 30 min, 5% B. Detection by the DAD was performed at 280–320 nm. For the MS assay, a 6120 Agilent Quadrupole LC/MS (Agilent Technologies, Santa Clara, CA, USA) coupled to the HPLC and equipped with an electrospray ionization interface in negative ionization mode, was used with the following conditions: drying gas flow (N_2_), 13.0 L min^−1^; nebulizer pressure, 40 psi; gas drying temperature, 350 °C; capillary voltage, 3500 V. The data analysis was performed using OpenLab CDS ChemStation Edition software (C.01.07, Agilent Technologies). Characterization of the PCs was based on retention time and mass spectrometric data. The compounds were first detected by a single MS scanning in the m/z range from 100 to 1100 followed by a targeted search based on the peaks showing major signals in the UV–Vis chromatograms.

### 2.5. In Vitro Digestion and Bioaccessibility (%) of the Mango-Based Bars

**Figure 1:**
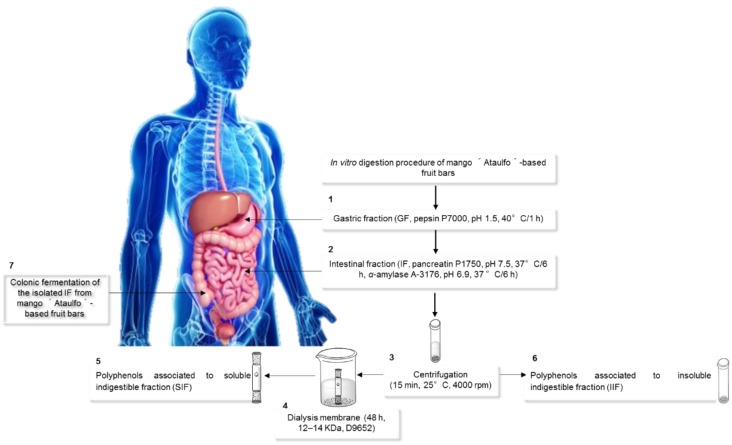
In vitro digestion of the mango bars. Step 1, gastric fraction (GASF) (pepsin); Step 2, intestinal fraction (IntF) (pancreatin and amylase); Step 3, centrifugation to separate supernatants and residues; Step 4, dialysis for 24–48 h; Step 5, non-bioaccessible PCs associated with soluble indigestible fraction (SIF); Step 6, non-bioaccessible PCs associated with insoluble indigestible fraction (IIF); and Step 7, colonic fermentation of the isolated indigestible fraction (IF) from mango bars.

The freeze-dried samples were subjected to in vitro digestion as previously described [23] (Figure 1). Briefly, the samples were subjected to hydrolysis with pepsin (300 mg/mL in HCl-KCl buffer, P-7000, Sigma-Aldrich, St. Louis, MO, USA) at pH 1 for 1 h at 37 °C. The PCs released after this stage were considered part of the gastric fraction (GasF) (Figure 1, Step 1). Immediately after the gastric phase, the samples were further digested in a simulated intestinal phase with a pancreatin solution (5 mg/mL in phosphate buffer, pH 6.9, 6 h, 37 °C) (P-1750, Sigma Aldrich, St. Louis, MO, USA) containing α-amylase (5 mg/mL in phosphate buffer, 16 h, 37 °C) (A-6255, Sigma Aldrich, St. Louis, MO, USA). The PCs released after this stage were considered part of the intestinal fraction (IntF) (Figure 1, Step 2). After both digestion phases, the samples were centrifuged (Figure 1, Step 3) to separate the soluble and insoluble indigestible fractions. The supernatant was dialyzed (D9652, 12–14 kDa, Sigma Aldrich, St. Louis, MO, USA) for 48 h to simulate passive absorption (Figure 1, Step 4). After dialysis, the TSP content and AOX associated with the soluble indigestible fraction (SIF) were determined (Figure 1, Step 5). The centrifugation residue was used to determine the TSP content associated with the insoluble indigestible fraction (IIF) after an aqueous-organic extraction [18] (Figure 1, Step 6). The PCs associated with the SIF and IIF correspond to the fraction that is non-bioaccessible in the small intestine. The different fractions, namely, GasF, IntF, SIF, and IIF, were assessed for TSP and AOX as described above. The different fractions were injected in an HPLC-DAD-MS to identify the PCs present in each fraction. The in vitro bioaccessibility percentage (%BA) of the PCs was determined using the following Equation (1):

(1) $$\%\;BA\frac{\left(PC\text{-}IntF\right)-\left(PC\text{-}SIF\right)}{\left(PC\text{-}IntF\right)+\left(PC\text{-}IFF\right)}\times 100$$

where, PC-IntF represents the PCs associated with the intestinal fraction, PC-SIF are the PCs associated with the soluble indigestible fraction, and PC-IIF are the PCs associated with the insoluble indigestible fraction [23].

### 2.6. Isolation and Quantification of Indigestible Fraction (IF), and Its In Vitro Colonic Fermentation in Mango-Based Bars

To evaluate the content of the indigestible fraction after in vitro gastrointestinal digestion, the samples were submitted to physiological digestion conditions of the human upper digestive tract, according to the protocol by Saura-Calixto et al. [24] for assessing the soluble indigestible fraction (SIF), insoluble indigestible fraction (IIF), and total indigestible fraction (TIF) of the bars. The TIF to be submitted to the in vitro fermentation process was isolated following the protocol proposed by Tabernero et al. [25]. The TIF isolated was fermented according to Zamora-Gasga et al. [26] (Figure 1, Step 7). Briefly, three healthy volunteers donated fresh fecal samples for preparing a pool. The culture media was reduced using an anaerobic chamber for 12 h before the fermentation. The fecal pool aliquots were diluted (1:10, w/v) with phosphate buffer (0.1 mol/L, pH 7) and homogenized. The fecal suspension was added in disposable tubes with 9 mL of nutritive medium, and 100 mg of the isolated IF. Besides the samples, two different controls were conducted in parallel: (a) raffinose, used as a fermentable carbohydrate that produces SCFA (positive control), and (b) the culture media inoculated with fecal suspension, which was used as negative control. All incubations were performed in triplicate. The samples and controls were collected after 6, 12, 24, and 48 h and centrifuged (3500× *g*, 15 min, 4 °C) (Hermle Z 323 K; Wehingen, Germany). The supernatants were divided for the analytical assays (pH, TSP, identification of PC and their metabolites by HPLC-DAD-MS, as described before and SCFA quantification by GC-MS).

### 2.7. SCFA Quantification by GC-MS Analysis

The evaluation of SCFA concentrations was performed according to Zamora-Gasga et al. [27]. The volatile constituents were analyzed with an Agilent 5977A mass selective detector coupled to an Agilent 7890B gas chromatograph (Agilent Technologies), equipped with a DB-5MS capillary column (60 m × 250 m × 0.25 m, Agilent Technologies). Sample quantification was obtained by means of acetic, propionic, and butyric acid standard curves (Sigma-Aldrich). The identification of the volatile components was completed by comparing the mass spectra of the samples with the data system library MSD ChemStation software (Agilent G1701EAversion E.02.00.493). The results were expressed in mmol/L produced per 100 mg DW of substrate.

### 2.8. Statistical Evaluation

All analyses were performed in triplicate. Mean values and standard deviations or standard error depending of the case from each determination were calculated. Data were subjected to a one-way ANOVA and significant differences were reported using LSD Fisher’s test with Statistic 8.0 Release for Windows software (Stat Soft. Inc., Tulsa, OK, USA) with a significance level of α = 0.05.

## 3. Results and Discussion

### 3.1. Nutritional Composition, PCs, and AOX in Mango-Based Bars

**Figure 2:**
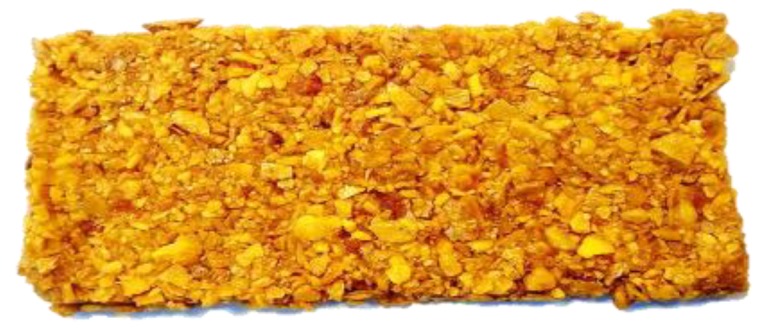
Mango-based bar prepared with peel and paste.

Figure 2 shows the bar prepared with the pulp and peel of mango. The bars were tasted by a non-trained group of panelists, with good general acceptance. The product was considered delicious and all panelists expressed that they would consume it regularly. The taste was similar to fresh mango and the most positively mentioned characteristics were color and aroma.

The nutritional composition of the bars is shown in Table 1. Moisture contents were lower than 10%. Moisture values of 15.73% and 18.26% were reported for fruit bars developed from nabtat ali and sukkari, respectively [28]. Such moisture value is a predictor of a low Aw, which indicates a potential high stability feature for the mango-based bar. The protein content was very low as compared with snack bars prepared, for instance, with apple puree (2.74%–3.66%) [29]. Similarly, the fat content was lower than those reported for other fruit bars [28,29], which may reflect that, here, the studied bars were developed with the whole fruit only, without added fat or additives. The mineral content, represented by the ash portion, was higher than 2%. Mango is considered a source of minerals such as calcium, magnesium, phosphorus, sodium, and iron [30]. Total soluble carbohydrates were the main component in the mango-based bar and sucrose is one of the most common carbohydrates in mango pulp. The monosaccharides reported for mango are derived from the hydrolysis of starch, pectins, and other cell wall constituents during the ripening process, which yields mainly glucose, fructose, and other carbohydrates, such as rhamnose, galactose, and arabinose [31].

The TDF content of the bars was 31.8%, which is higher than the value reported for Ataulfo mango pulp (17.65%–24.70%) [32], and a mango paste by-product (14.97%), but lower than in Ataulfo mango peel (41.34%) [9]. This was an expected result, considering that the studied bars were prepared with a blend of mango peel and pulp. The mango bars showed higher TDF values as compared with sukkari fruit bars and nabtat ali bars (5.51% and 4.43%, respectively) [28]. Pectins and other polysaccharides such as galactans and arabinogalactans are part of the SDF in mango pulp and peel [33]. The presence of peels in the bars contributes cellulose, lignin, and hemicelluloses (arabinoxylans and arabinogalactan), which are important parts of IDF in fruits [34]. The SDF and IDF contents were similar in the mango bars, i.e., the SDF/IDF ratio was about 1:1. It has been suggested that a 0.3:0.7 value is a good proportion for a DF source [35]. Since the ratio recorded here was remarkably higher, the DF quality of these bars may be considered to be good. The amount of TDF in a 30 g portion of mango bar was 9.5 g, which represents about 35% of the recommended daily intake in a healthy diet [36].

TSP content in the fruit-based bars developed in this work was higher than in similar products reported elsewhere [28,29]. For instance, sapodilla- (*Manilkara zapota* L.) containing bars, added with pectin in different proportions, contained 176 mg GAE/100 g [37]. Regarding the AOX in the mango-based bars, the ABTS value was higher than FRAP. This can be related to the fact that ABTS can measure lipophilic antioxidant compounds, such as carotenoids or phytoesterols, resulting in higher values [38]. Although the content of carotenoids was not quantified, the presence of these compounds should not be ignored. In that sense, all-trans-βcarotene, 9-cis-β-carotene, all-trans-lutein, and 13-cis-β-cryptoxanthin were identified in paste and peel of mango cv. Ataulfo obtained by hot-air drying. The authors reported a total carotenoid content of 29.57 and 67.82 g/kg DW for paste and peel, respectively [39]. Recently, a β-carotene content of 99.10 micrograms/gram was reported in an extruded snack based on taro flour (*Colocasia esculenta* L.) enriched with 7.97 g/100 g mango pulp var Manila [40].

On the basis of this, the bars studied here may be considered relatively rich in carotenoids. Hydrolyzable polyphenols (HP) are polymers of gallic acid that occur mostly in the peel of fruits [41]. Since it has been reported [42] that mango peels contain mainly HP, it is tempting to state that the presence of these PCs improves the composition of the mango-based bar.

### 3.2. PCs Identified by HPLC-DAD-MS in Mango-Based Bars

Table 2 shows the PC profile for mango-based bars, where mainly four phenolic groups were identified: phenolic acids, cinnamic acids, one flavonoid, and xanthones. Gallic acid has been previously reported as an important constituent of Ataulfo mango, and its presence was clearly detected in the bars. Ferulic, coumaric, and caffeic acids were the cinnamic acids identified, where ferulic was the major compound. A similar pattern was reported for “Ataulfo” mango peel and pulp [9,41]. Hydroxicinnamic acid derivatives have been reported in the fiber fraction of different fruits, forming cross-links with cell wall polysaccharides [34]. The other major member of the flavonol group identified in the bars was quercertin, which agrees with reports indicating the presence of a similar group of flavonoids in mango peels [43]. Finally, two xanthones, mangiferine and mangiferine gallate, were also identified in the mango bars, with mangiferine as the predominant compound. This compound is present in mango peel and is recognized for its functional properties, such as anti-inflammatory effect and protective action against damage caused by oxidative stress in some tissues [44,45]. PCs are recognized by their potential role in the prevention of degenerative diseases and their antimicrobial properties, anticancer activity, and their apparent inverse relationship with cardiovascular problems [46].

### 3.3. Release of PCs in GasF, IntF, and %BA in Mango-Based Bars

The interest in the PCs bioavailability goes in parallel with the identification and quantification of these components in foods. The PCs can be released in the gastrointestinal tract during the digestion process and thus exert beneficial effects on health. In vivo trials have several limitations, however, these assays are considered a valid approach to the in vivo conditions and can provide a foundation for further exploration via in vivo assays [36]. Table 3 shows the TSP content in the mango-based bars after the GasF and IntF. These data indicate that the TSP content of the samples after GasF was slightly higher (~9%) than that observed after the IntF. Overall, the in vitro simulated digestion had a significant effect on the PCs content. During the GasF, PCs linked to polysaccharides in the plant cell wall, as well as flavonoids in the cytosol and in the endoplasmic reticulum, can be released [47]. Additionally, the low pH and pepsin activity release most of the PCs that are covalently linked to cell walls [10]. The AOX did not show a significant difference (*p* > 0.05) between the GasF and IntF fractions. The AOX of flavonoids can decrease after the simulated duodenal passage during in vitro intestinal digestion [48]. The AOX showed positive correlation with the TSP contents registered in the GasF and IntF. Other bioactive compounds, such as carotenoids, can be released during the gastrointestinal phase and can influence the AOX values [49].

The PCs identified during the GasF and IntF for mango bars exhibited a higher MS area than that reported in the aqueous-organic extract. This may be due to the low pH and pepsin action in the stage of gastric digestion, which may cause partial release of the PCs bound to the cell walls of the fruit matrix [50]. In the intestinal stage, the release of PCs is affected by changes in the pH and the action of pancreatin and α-amylase enzymes. It is known that both factors weaken the interactions between carbohydrates and PCs, which increases the PCs bioavailability [51].

The PCs identified in the GasF and IntF were similar to those found during the characterization of the mango-based bar, however, mangiferin gallate could not be identified after the digestion process, while two additional PCs, hydroxicinnamic acid and kaempferol, were identified after digestion. These observations indicate that mangiferine gallate can undergo hydrolysis during the gastric digestion which leads to complete release of the mangiferin molecule. The release of other PCs, such as hydroxicinnamic acid and kaempferol, needs the action of intestinal enzymes. Recent in vitro digestion studies with strawberry and the tropical juçara fruit, showed that the profile of the PCs varies during the different digestion steps [52,53]. All these compounds have been previously identified in mango pulp [41], while mangiferin has been identified as the main bioactive compound in mango peel. Lower contents of the different compounds were recorded in the IntF as compared with the GasF, however, the released PCs are potentially available for absorption at the end of the intestinal digestion [10].

The bioaccessibility (BA) of PCs was calculated based on the release of PCs in the different stages of in vitro gastrointestinal digestion and the PCs content associated with SIF and IIF. The %BA value, which indicates the PCs that are potentially bioaccessible to be absorbed by the enterocytes, was 53.78% for the mango bar (Table 3). The bioaccessibility of PCs in some plant-based foods has been reported earlier, e.g., mango by-products (peel and paste), roselle calyces, decoction residues (*Hibiscus sabdariffa* L.), and hot peppers (*Capsicum annum* L.) [9,54,55]. The bioaccessibility value obtained in this study for the mango bar was close to that obtained for different by-products from the industrial processing of mango. The results showed that the PCs that were not linked to the indigestible fractions (dietary fiber) could be released during in vitro digestion. This suggests that some of the PCs in mango are not bound to the food matrix which makes these PCs readily available for intestinal absorption [10,56].

### 3.4. PCs Bound to the Indigestible Fraction (IF) Isolated from Mango-Based Bars

Table 4 shows the content of TIF (38.72 ± 2.18 g/100 g), which is the sum of the SIF (24.44 ± 0.85 g/100 g) and IIF (14.28 ± 1.35 g/100 g). The high values of SIF and IIF can be attributed to the fact that the bar is made with the pulp and peel of Ataulfo mango, which have been previously reported to be important sources of IF and associated PCs [57,58]. These components resist the gastrointestinal digestive processes, and together with the PCs present in the matrix of the fruit, can reach the colon and serve as a substrate for the microbiota [59].

PCs can associate with components of DF, binding onto their surface. This interaction can decrease the bioaccessibility of PCs and the unabsorbed PCs fraction can be “carried” by fibers and thus reach the colon, where they may be also metabolized by the gut microbiota [60]. Table 4 shows the contents of PCs bound to SIF and IIF. In the in vitro system used here, the PCs associated to the SIF, i.e., those which were not able to diffuse across the dialysis membrane, are PCs that can be associated with soluble DF from the food matrix or have high molecular weights [61]. The content of PCs associated with IIF (60.98 ± 0.14 g GAE/100 g), was higher than that found in the SIF (41.86 ± 0.10 g GAE/100 g), which indicates that a large portion of the IIF is constituted by bioactive compounds. This fraction corresponds to the residue of the food matrix and contains IIF, HP, and other insoluble compounds [36] (Table 4). Our results agree with those reported by Blancas-Benítez et al. [9], where hydroxycinnamic and ferulic acids were identified as the PCs associated with the dietary fiber of peel and paste of Ataulfo mango, while coumaric and gallic acid were mainly present in the paste, and caffeic acid was associated with the peel. Regarding flavonoids, quercetin and kaempferol were associated with the IF isolated from the mango bar, similarly to the xanthone, mangiferin (Table 4). The PCs associated with the IF, besides being potential substrates for the microbiota, and thus capable to exert beneficial actions, may be bioconverted to other hydroxyphenolic acids or derivatives.

Mangiferin has anti-inflammatory potential. Jeong et al. [62] reported that mangiferin ameliorated induced colitis in mice by inhibiting inflammatory signal pathways. Quercetin and caffeic acid have been shown to be capable of modifying the gut microbiota by stimulating the proliferation of *Bifidobacteria* and decreasing the ratio of *Firmicutes* to *Bacteroidetes* in vitro [60]. In addition, gallic acid may inhibit the growth of pathogens like *Clostridium perfringens* and *C. difficile* [59]. On the other hand, kaempferol has been shown to modulate the peroxisome proliferator-activated receptor-γ (PPR-γ), which is involved in oxidative stress response and neuro-inflammation.

According to the results summarized in Table 4, the AOX (ABTS and FRAP) of PCs measured in the IIF did not show significant difference (*p* > 0.05) from that recorded in the SIF. The percentage of non-bioaccessible PCs fraction (SIF and IIF) was 46.22%. This result agrees reasonably with the general estimation made by Saura-Calixto et al. [63], who stated that 42% of the dietary PCs might reach the colon.

### 3.5. Changes in pH and AOX during In Vitro Colonic Fermentation

**Figure 3:**
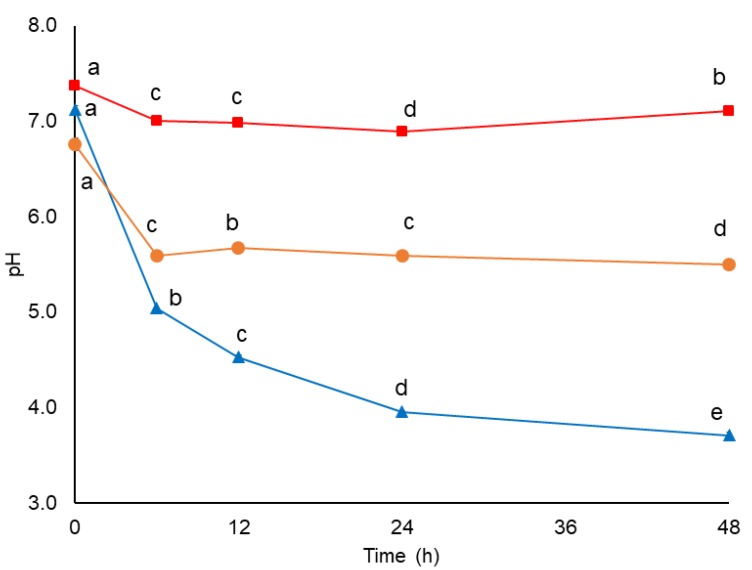
pH changes during in vitro colonic fermentation of negative control (■), raffinose (▲), and indigestible fraction of (●) mango bar. Values are means ± Standard Error of the Mean (SEM) (*n* = 3). Different letters show significant difference between each fermentation time per sample using two-way ANOVA/Fisher’s LSD test, *p* < 0.05.

The pH data were collected during 48 h of in vitro colonic fermentation of the total indigestible fraction isolated from mango-based bar (TIF-MB) (Figure 3). The starting pH values (before fermentation) ranged between approximately 6.76 and 7.37, and decreased in both TIF-MB and the raffinose reference already after 6 h fermentation, a change that was related to the production of acetic acid, the most abundant SCFA found in the fermented samples (Table 6). Acetic acid is not only beneficial as a relatively strong organic acid leading to pH reduction and inhibition of pathogens growth [64], but it is also involved in lipid metabolism in the liver by directly up-regulating PPARα target genes, and therefore resulting in increased fatty acid oxidation and decreased hepatic lipid storage [65]. The magnitude of the pH value reduction was markedly different depending on the fermentation time (*p* < 0.05) and the pH variations (pH ∆_0h–48h_) were 0.26, 3.42, and 1.26 for negative control, raffinose, and TIF-MB, respectively. A low pH favors the absorption of minerals such as calcium and magnesium. It also reduces the toxic side effects of luminal ammonia by promoting its absorption as NH_4_^+^ by the bacterial mass and its removal through faeces instead of urine as NH_3_ [11]. However, Ye et al. [66] indicated that the lower pH and variations in the relative abundance of certain microbiota genera may contribute by changing the expression of colonic cytokines, such as IL-1β, IFN-γ and IL-10, promoting epithelial inflammation in the large intestine.

### 3.6. Short Chain Fatty Acids (SCFA) during In Vitro Fermentation

The SCFA concentration in the intestine (20–140 mM) depends on the composition of the microbiota, the intestinal transit time, the flow of SCFA by the metabolism of the colonic microbiota, and the fiber content and composition of the host’s diet [67]. In this study, the SCFA levels increased significantly over time in TIF-MB (Table 5), by seven-fold during the first 24 h. The TIF-MB resulted in ~140 mmol/L levels of acetate at the end of fermentation (48 h). Acetic acid concentrations were significantly higher for the raffinose reference at all times as compared with TIF mango-based bar by 3.6- to 8.9-fold at 6 h and 48 h, respectively (*p* < 0.05). Acetic acid is produced by most enteric bacteria but also by acetogenic bacteria (which produce three acetate molecules from a glucose molecule), such as *Blautia hydrogenotrophica,* through the Wood-Ljungdahl pathway. Non-acetogenic anaerobes must eliminate reducing equivalents by forming other products besides acetic acid, including succinic, propionic, butyric, formic, D-lactic, and L-lactic acids, as well as ethanol [68]. On the other hand, propionate levels increased significantly (*p* < 0.05) for the two substrates throughout the experimental period, although TIF in the mango-based bar elicited significantly higher levels at 24 h as compared with other fermentation times (Table 5). With the exception of the 6 h and 12 h samples, raffinose led to higher propionic acid levels (~52 mmol/L) throughout the 48 h of fermentation. These results suggest a potential beneficial effect of mango-based bar consumption. In this sense, propionic acid derived from the intake of inulin-type fructans reduces hepatic BaF3 cells growth through a cAMP level-dependent pathway. In addition, the activation of free fatty acid receptor 2 (FFA2), a Gi/Gq protein-coupled receptor also known as GPR43 that binds propionate, lessens the proliferation of BaF3 and other human cancer cell lines [69]. Finally, butyrate levels in TIF-MB increased during the entire experimental fermentation period, from non-detectable levels at 6 h to 9.71 mmol/L at 48 h (Table 5). Similar butyric acid concentrations were observed for raffinose and TIF-MB (*p* > 0.05). Butyric acid possesses a remarkable variety of antineoplastic properties and promotes colon health. It maintains the integrity of the mucosa and suppresses inflammation and carcinogenesis through effects on immunity, gene expression, and epigenetic modulation [70]. *Eubacterium rectale/Roseburia* spp. (estimated at 2%–15% of total bacteria) within *Lacnospiraceae* (clostridial group XIVa), and *Faecalibacterium prausnitzii* within *Rumincoccaceae* (clostridial group IV) are producers of butyric acid [71].

### 3.7. Bioconversion of PC during In Vitro Colonic Fermentation of IF Isolated from Mango Bar

The PCs released during the in vitro colonic fermentation of IF from mango bar are summarized in Table 6. Significantly different patterns (*p* < 0.05) were observed among the various PCs metabolites identified. Gallic acid was identified during the whole in vitro colonic fermentation, while hydroxycinnamic acids (ferulic, chlorogenic and coumaric acid) were no longer detected after 12 h. In accordance with this observation, chlorogenic acid has been reported as a rapidly fermentable substrate, being degraded within the first few hours [72]. Several studies suggest that gallic acid decreases tumor size by attenuating the expression of cytokines [59], while ferulic acid improved kidney structure and function in hypertensive rats [73].

The presence of mangiferin and norathyriol was detected after 6 h and 12 h fermentation. Li et al. [74] reported that norathyriol is one of the main compounds from mangiferin metabolism. Lin et al. [75] suggested that this metabolite possesses potential antihyperuricaemic activity related to the inhibition of uric acid production by targeting the secretory organic anion transporter 1 (OAT1).

**Figure 4:**
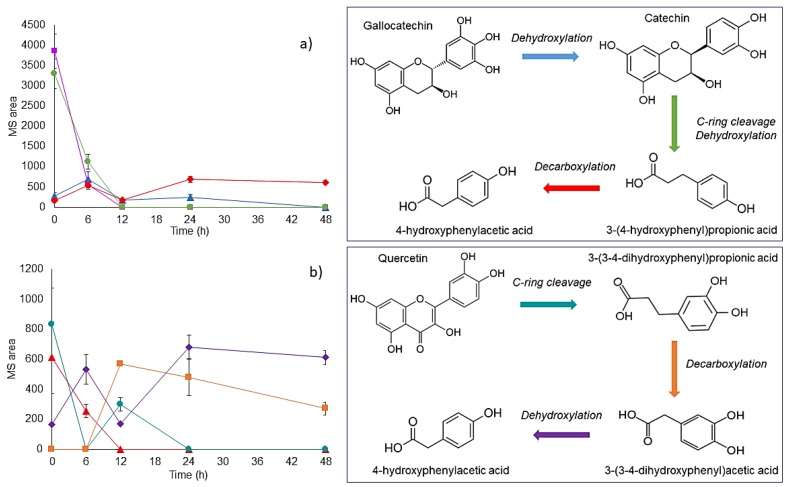
(**a**) Biotransformation of (■) gallocatechin to (▲) catechin, (●) 3-(4-hydroxyphenyl) propionic acid and (◆) 4-hydroxyphenylacetic acid during 48 h of colonic fermentation. (**b**) Biotransformation of (▲) quercetin to (●) 3-(3,4)-dihydroxyphenylpropionic acid, (■) 3,4-dyhydroxyphenylacetic acid and (◆) 4-hydroxyphenylacetic acid, during 48 h of colonic fermentation.

Regarding flavonoids, gallocatechin was totally bioconverted after 6 h of fermentation. On the other hand, Catechin levels increased after 6 h of fermentation, which suggests that it can be produced by dehydroxylation of gallocatechin [15]. Catechin can be further bioconverted to 3-(4-hydroxyphenyl) propionic acid by dehydroxylation and C-ring cleavage. After 12 h of fermentation, 3-(4-hydroxyphenyl) propionic acid was no longer detected; this compound may be a precursor metabolite of 4-hydroxyphenylacetic acid by decarboxylation. An increase in 4-hydroxyphenylacetic acid relative abundance was observed at 6 h and 24 h of fermentation, without significant differences (*p* > 0.05) at 48 h (Figure 4a). In agreement with this result, Low et al. [76] reported that 3-(4-hydroxyphenyl)propionic acid decreased in a sustained manner during the first 24 h of fermentation of masticated mango, disappearing completely after 48 h. The main metabolite detected here after 4 h of fermentation was 4-hydroxyphenylacetic acid which can be produced through the decarboxylation and subsequent dehydroxylation of 3-(4-hydroxyphenyl) propionic acid.

Quercetin was no longer present after 6 h of fermentation. This flavonoid may be biotransformed to 3-(3,4)-dihydroxyphenylpropionic acid by a C-ring cleavage. Then, 3-(3,4)-dihydroxyphenylpropionic acid can be a precursor metabolite of decarboxylation products, such as 3,4-dihydroxyphenyl acetic acid. As shown in Table 5, 3-3,4-dihydroxyphenyl acetic acid was detected after 12 h of fermentation without significant differences along the 12–48 h period (*p* > 0.05). 4-Hydroxyphenylacetic acid is proposed as a metabolite produced by dehydroxylation of 3,4-dihydroxyphenyl acetic acid (Figure 4b). In this regard, hydroxyphenylacetic acids have been characterized as specific metabolites produced during the colonic degradation of quercetin and its glycosylated derivatives, through C-ring cleavage in 3-(3,4)-dihydroxyphenyl) propionic acid and subsequent degradation to 3-4-dihydroxyphenylacetic acid [77]. Several modes of C-ring fission of flavonol are possible, which can finally yield the detected product, 3,4-dihydroxyphenylacetic or 3-(3-hydroxyphenyl) propionic acid, corresponding to fission between the oxygen of C2 and C3–C4 ring and between the oxygen of C2 ring and C4-A ring, respectively [78]. Particularly, 3,4-dihydroxyphenylacetic acid, which is a metabolite of the neurotransmitter dopamine, inhibits the formation of advanced glycosylation end products and is effective in preserving cultured neuronal cells, preventing their damage by oxidative stress [79].

## 4. Conclusions

Mango-based bars contained high concentrations of PCs, which was in agreement with their high AOX. The main PCs identified during the gastrointestinal digestion of the bars were quercertin, gallocatequin, and mangiferin. The potential health-beneficial effects of PCs associated to DF can be better understood through the study of their behavior during gastrointestinal digestion and colonic fermentation. The bioaccessibility of the PCs in the bars was ca. 54%, meaning that 46% of PCs may reach the colon and can be bioconverted into different hydroxyphenolic acids. Although further studies are required to establish the in vivo beneficial effects of mango-based bars and other fruit-based products, these food items are promising means for a relatively cheap and sustainable increase of the intake of DF and related bioactive PCs.

## Figures and Tables

**Table 1:** Nutritional composition, total soluble polyphenols (TSP), antioxidant capacity (AOX; ABTS, FRAP), and hydrolyzable polyphenols (HP) of mango-based bar. ^1^

Nutritional Composition (g/100 g DW)	
Moisture	8.33 ± 0.22
Protein ^2^	1.69 ± 0.13
Fat	0.45 ± 0.01
Ash	2.95 ± 0.09
TSC ^3^	51.98 ± 0.61
TDF ^4^	31.85 ± 0.22
SDF	14.38 ± 0.15
IDF	16.94 ± 0.11
TSP (g GAE/100 g sample DW)	14.35 ± 0.70
AOX (μmol TE/g sample DW)	
ABTS	314.00 ± 1.43
FRAP	201.03 ± 20.1
HP (g GAE/g sample DW)	5.43 ± 0.26

^1^ Values represent mean ± standard deviation (*n* = 3), ^2^ N × 6.25 conversion factor, ^3^ TSC: total soluble carbohydrates, and ^4^ TDF: total dietary fiber as the sum of soluble dietary fiber (SDF) + insoluble dietary fiber (IDF).

**Table 2:** Total soluble polyphenols profile in mango-based bar. ^1^

Compound	RT (min)	M/Z (-)	Relative Abundance (%) ^2^
**Phenolic acids**			
Gallic acid	3.74	169	0.46
**Cinnamic acids**			
Coumaric acid	3.839	168	2.10
Ferulic acid	4.043	193	9.72
Caffeic acid	4.153	179	0.61
**Flavonoids**			
Quercetin	3.914	301	2.71
**Xanthones**			
Mangiferin gallate	4.287	573	3.27
Mangiferin	14.269	421	81.13

^1^ Values represent mean ± standard deviation (*n* = 3) and ^2^ relative abundance (%) calculated from arbitrary units from MS area.

**Table 3:** Release of total soluble polyphenols (TSP), phenolic compounds (PCs) profile, and antioxidant capacity (AOX; ABTS, FRAP) in gastric fraction (GasF), intestinal fraction (IntF), and bioaccessibility (%) upon in vitro digestion of mango-based bar.

	g/100 g DW
**GasF (g/100 g DW)**	
TSP (g GAE/100 g DW)	16.79 ± 0.03 ^a^
**PCs profile (MS area) ^1^**	
Gallic acid	1.90
2-Hydroxycinnamic acid	0.30
Ferulic acid	2.33
Caffeic acid	0.10
Mangiferin	8.67
Kaempferol	84.30
p-Coumaric acid	1.82
Quercetin	0.58
**AOX (μmol TE/100 g DW)**	
ABTS	470.77 ± 0.02 ^c^
FRAP	22.73 ± 0.08 ^d^
**IntF (g/100 g DW)**	
TSP (g GAE/100 g DW)	15.32 ± 0.19 ^b^
**PCs (relative abundance% ^1^)**	
Gallic acid	5.57
2-Hydroxycinnamic acid	21.72
Ferulic acid	1.08
Caffeic acid	0.65
Mangiferin	44.16
Kaempferol	18.59
p-Coumaric acid	5.18
Quercetin	3.05
**AOX (μmol TE/100 g DW)**	
ABTS	469.98 ± 0.01 ^c^
FRAP	14.54 ± 0.08 ^d^
**Bioaccessibility of PCs (%)**	53.78 ± 0.03

^1^ Relative abundance (%) calculated from arbitrary units from MS area, values represent the mean ± standard deviation (*n* = 3), different letters in the same column indicate a significant difference in each assay (*p* < 0.05), ND = not detected. $\mathrm{Bioaccessibility}\%=\frac{\left(\mathrm{PC-IntF}\right)-\left(\mathrm{PC-SIF}\right)}{\left(\mathrm{PC-IntF}\right)+\left(\mathrm{PC-IIF}\right)}\times \;100$, PC-IntF = the PCs released in the intestinal fraction, PC-IIF = the PCs associated with insoluble indigestible fraction, and PC-SIF = the PCs associated with soluble indigestible fraction.

**Table 4:** Total, soluble, and insoluble indigestible fraction (TIF, SIF, and IIF) content, total soluble polyphenols (TSP), antioxidant capacity (AOX; ABTS, FRAP), phenolic compounds (PCs) profile, and non-bioaccessible content upon in vitro digestion of the mango-based bar.

**TIF (g/100 g DW)**	38.72 ± 2.18
**SIF (g/100 g DW)**	24.44 ± 0.85
TSP (g GAE/100 g DW)	41.86 ± 0.10 ^a^
**PCs profile (relative abundance% ^1^)**	
Gallic acid	9.51
2-Hydroxycinnamic acid	19.98
Ferulic acid	ND
Caffeic acid	0.32
Mangiferin	16.78
Kaempferol	50.73
p-Coumaric acid	1.25
Quercetin	1.43
**AOX (μmol TE/100 g DW)**	
ABTS	117.50 ± 0.02 ^a^
FRAP	6.15 ± 0.06 ^a^
**IIF (g/100 g DW)**	14.28 ± 1.35
TSP (g GAE/100 g DW)	60.98 ± 0.14 ^b^
**PCs profile (relative abundance% ^1^)**	
Gallic acid	ND
2-Hydroxycinnamic acid	ND
Ferulic acid	5.65
Caffeic acid	ND
Mangiferin	4.86
Kaempferol	85.34
p-Coumaric acid	1.61
Quercetin	2.54
**AOX (μ** **mol TE/100 g DW)**	
ABTS	118.21 ± 0.01 ^b^
FRAP	11.87 ± 0.01 ^b^
**Non-bioaccessible PCs fraction (%)**	46.22 ± 0.03

^1^ Relative abundance calculated from arbitrary units from MS area, values represent the mean ± standard deviation (*n* = 3), different letters in the same column indicate significant differences on each assay, ND = not detectable.

**Table 5:** Concentration of short chain fatty acids (SCFA) produced during the colonic fermentation of total indigestible fraction of mango-based bar (TIF-MB) and positive control (raffinose). ^1^

SCFA	Time (h)	Raffinose mmol/L	TIF-MB mmol/L
Acetic acid	6	62.60 ± 4.31 ^a,D^	19.75 ± 4.78 ^a,B^
	12	193.84 ± 30.74 ^a,C^	25.68 ± 1.45 ^b,B^
	24	465.54 ± 48.85 ^a,B^	146.62 ± 52.87 ^b,A^
	48	1295.40 ± 170.86 ^a,A^	144.82 ± 25.61 ^b,A^
Propionic acid	6	ND ^a,D^	ND ^a,C^
	12	3.21 ± 0.84 ^a,C^	2.38 ± 0.89 ^a,B^
	24	27.05 ± 4.82 ^a,B^	11.10 ± 2.71 ^b,A^
	48	52.31 ± 1.05 ^a,A^	5.13 ± 1.50 ^b,A,B^
Butyric acid	6	0.12 ± 0.00 ^a,C^	ND ^a,D^
	12	1.62 ± 0.24 ^a,B^	0.50 ± 0.03 ^b,C^
	24	4.38 ± 0.70 ^a,A^	3.04 ± 0.06 ^a,B^
	48	5.24 ± 1.94 ^a,A^	9.71 ± 1.32 ^a,A^

^1^ Values have been reported as mean ± standard error of three replicates. Different lowercase letters indicate significant differences in rows among substrates for a time and different capital letters indicate significant differences in columns among time for a substrate using two-way ANOVA/Fisher’s LSD test, *p* < 0.05. ND = not detectable.

**Table 6:** Phenolic compounds identified on in vitro colonic fermentation of indigestible fraction of mango bars by HPLC-DAD-MS.

Compound	RT (min)	m/z (-)	Relative Abundance ^1^			
			6 h	12 h	24 h	48 h
**Hydroxybenzoic acids**						
Gallic acid	6.33	169	77.7 ^a^	18.1 ^b^	3.0 ^c^	1.2 ^c^
**Hydroxycinnamic acids**						
Ferulic acid	3.91	193	93.3 ^a^	6.7 ^b^	ND	ND
Coumaric acid	4.33	163	ND	100.0	ND	ND
Chlorogenic acid	4.38	353	79.4 ^a^	20.6 ^b^	ND	ND
**Flavonoids**						
Quercetin	19.64	301	100.0	ND	ND	ND
Catechin	4.59	289	61.7 ^a^	16.3 ^b^	22.0 ^b^	ND
Galocatechin	3.94	305	100.0 ^a^	ND	ND	ND
Galocatechin galate	12.41	457	93.4 ^a^	6.6 ^b^	ND	ND
**Xanthones**						
Mangiferin	14.39	421	68.5 ^a^	31.5 ^b^	ND	ND
Norathyriol	11.34	259	87.7 ^a^	12.3 ^b^	ND	ND
**Hydroxyphenolic acids**						
3-(3,4)-Dihydroxyphenylpropionic acid	5.25	181	ND	100.0	ND	ND
3-(4-Hydroxyphenyl)propionic acid	4.01	165	100.0	ND	ND	ND
3,4-Dihydroxyphenylacetic acid	10.81	167	ND	43.2 ^a^	36.3 ^a^	20.5 ^a^
4-Hydroxyphenylacetic acid	12.23	151	27.5 ^a^	8.8 ^b^	33.8 ^a^	29.9 ^a^
4-Hydroxybenzoic acid	11.29	137	24.5 ^a^	38.4 ^a^	37.1 ^a^	ND

^1^ Relative abundance (%) calculated from arbitrary units from MS area, different lowercase letters in the same row indicates significant differences (*p* < 0.05). ND = not detectable.
